# Ten-Year Trends in Fiber and Whole Grain Intakes and Food Sources for the United States Population: National Health and Nutrition Examination Survey 2001–2010

**DOI:** 10.3390/nu7021119

**Published:** 2015-02-09

**Authors:** Carla R. McGill, Victor L. Fulgoni, Latha Devareddy

**Affiliations:** 1 Healthy Science Communications, LLC, Sarasota, FL 34234, USA; 2 Nutrition Impact, LLC, Battle Creek, MI 49014, USA; E-Mail: vic3rd@aol.com; 3 The Coca Cola Company, Atlanta, GA 30313, USA; E-Mail: ldevareddy@coca-cola.com

**Keywords:** NHANES, fiber, whole grain, trends, food sources

## Abstract

Current U.S. dietary guidance includes recommendations to increase intakes of both dietary fiber and whole grain (WG). This study examines fiber and WG intakes, food sources and trends from 2001 to 2010 based on National Health and Nutrition Examination Survey (NHANES) data for children/adolescents (*n* = 14,973) and adults (*n* = 24,809). Mean fiber intake for children/adolescents was 13.2 (±0.1) g/day. Mean fiber intake for adults 19–50 years (y) was 16.1 (±0.2) g/day and for adults 51+ was 16.1 (±0.2) g/day. There were significant increases in fiber intake from 2001–2010 for children/adolescents and for adults 51+ y. Mean WG intake for children/adolescents was 0.52 (±0.01) oz eq/day. Mean WG intake for adults 19–50 y was 0.61 (±0.02) oz eq/day and for adults 51+ 0.86 (±0.02) oz eq/day. There were no significant changes in WG intake for any age group from 2001–2010. The main food groups contributing to dietary fiber intake for children/adolescents were vegetables (16.6%), grain mixtures (16.3%), other foods (15.8%) and fruits (11.3%). For adults 19+ y, the main sources of dietary fiber were vegetables (22.6%), other foods (14.3%), grain mixtures (12.0%) and fruits (11.1%). Major WG sources for children/adolescents included ready-to-eat cereals (RTEC) (31%), yeast breads/rolls (21%) and crackers and salty grain snacks (21%). The main sources of WG for adults 19+ were yeast breads/rolls (27%), RTEC (23%) and pastas/cooked cereals/rice (21%). Recommending cereals, breads and grain mixtures with higher contents of both dietary fiber and WG, along with consumer education, could increase intakes among the United States (U.S.) population.

## 1. Introduction

The 2010 Dietary Guidelines for Americans includes recommendations for intakes of both dietary fiber and whole grains (WG) [1]. Given that intakes of fiber in the United States (U.S.) were low enough to be of public health concern, fiber was identified as a nutrient of concern by the 2010 Guidelines. Most children, adolescents and adults do not consume the recommended amount of total dietary fiber [2]. Adequate intake (AI) values for fiber range from 19 to 25 g/day for children age 1 to 8 years (y), 26 to 38 g/day for children and adolescents aged 9 to 18 y and 21 to 38 g/day for adults 19 y or older [3]. Reicks *et al.* [4] recently reported that mean fiber intake for U.S. children/adolescents aged 2 to 18 y was 13.6 g/day and for adults 19+ y was 17.0 g/day based on the National Health and Nutrition Examination Survey (NHANES) 2009–2010 data. Recommendations for intake of WG have been included in the Dietary Guidelines since the year 2000, which stated “choose a variety of grains, especially whole grains” [5]. Both the 2005 and 2010 Dietary Guidelines recommended consumption of at least three ounce equivalents (oz eq) of WG daily and that at least half of all grains consumed be WG [1,6]. Based on NHANES 2009–2010 data, whole grain intakes were 0.57 oz eq/day for children/adolescents and 0.82 g/day for adults [4]. Recommendations for intakes of both dietary fiber and WG are based on low current intakes and associations with lower chronic disease risk, weight management benefits and better diet quality [1,7,8,9,10,11,12,13].

Based on data from NHANES 2003–2006, the top sources of dietary fiber for children/adolescents in the U.S. were fruit (10.4%) and yeast bread/rolls (10.3%) [14]. Top dietary fiber sources for adults from NHANES 2003–2006 were yeast bread/rolls (10.9%) and fruits (10.3%) [15]. Legumes, vegetables, fruit and WG were recommended by the 2010 Dietary Guidelines to help meet total dietary fiber recommendations [1]. Major sources of WG for the U.S. population were ready-to-eat cereals (RTEC), yeast breads/rolls, hot cereal and popcorn [4,16]. There has been increased visibility and consumer demand for WG foods since 2005 [17]. Recent survey data reported that fiber and WG content were top considerations for consumers when purchasing packaged foods [18]. Given the increased availability of fiber- and WG-containing foods in the U.S. over the past decade, there is little published data regarding food sources and trends in intakes over time.

The purpose of this study was to examine fiber and WG intakes, food sources and trends in intakes and sources over the 10-year period from 2001 to 2010 based on NHANES data for children, adolescents and adults. Consumption patterns of fiber and WG throughout the day by age were also examined to determine similarities and differences between fiber and WG intake patterns

## 2. Materials and Methods

### 2.1. Study Population

The continuous NHANES is a cross-sectional survey that collects data about the nutrition and health status of the U.S. population using a complex, multistage, probability sampling design [19]. The National Health and Nutrition Examination Survey is conducted in a noninstitutionalized, civilian U.S. population by the National Center for Health Statistics (NCHS). Participants of NHANES completed a comprehensive questionnaire assessing dietary behaviors, health history, socioeconomic status and demographic information at NHANES Mobile Examination Centers and in participant’s homes. The NCHS Research Ethics Review Board reviewed and approved all study protocols for NHANES 2001 to 2010. This was a secondary analysis of publicly available data, which lacked personal identifiers; therefore, this study did not require institutional review or approval.

Data are released in two-year increments, and for this analysis, data cycles from 2001 through 2010 were combined to increase sample size and assess 10-year trends in fiber and WG intakes. Data from children and adolescents 4–18 y (*n* = 14,973), adults 19–50 y (*n* = 13,268) and adults 51+ y (*n* = 11,541) were included. Analyses included only individuals with complete and reliable dietary records, as determined by the National Center for Health Statistics staff and excluded females who were pregnant or lactating [19].

Demographic information, including age, gender, race-ethnicity, socioeconomic status and education, used for covariates in the statistical analyses outlined below, was determined via interview [19].

### 2.2. Dietary Assessment

Trained interviewers conducted in-person 24-h dietary recalls using the U.S. Department of Agriculture’s (USDA’s) Automated Multiple-Pass Method [20,21]. Dietary data included detailed descriptions of all food and quantities eaten. Detailed descriptions of the dietary interview methods are provided in the NHANES Dietary Interviewer’s Training Manual [22]. Dietary intake data from Day 1 were used for analysis in this study. Total dietary fiber is a variable reported in NHANES and is based on values reported in the USDA’s Food and Nutrient Database for Dietary Studies (FNDDS). The MyPyramid Equivalents Database for USDA Survey Food Codes was used to calculate WG intake [23]. The MyPyramid Equivalents Database provides quantified measures of WG with separate tables based on the old and new (without bran) definitions for WG. The food data files contain the number of servings (oz eq) per 100 g of food for 32 MyPyramid food groups. Three of the food groups are WG, non-WG and total grain. Table 1 and Table 3 include those subjects who reported consuming fiber in the NHANES Day 1 dietary data. Table 2 and Table 4 include those subjects who reported WG consumption in the NHANES Day 1 dietary data. Eating occasion (breakfast, lunch, dinner and other eating occasions) was self-defined.

### 2.3. Statistical Analysis

**Figure 1:**
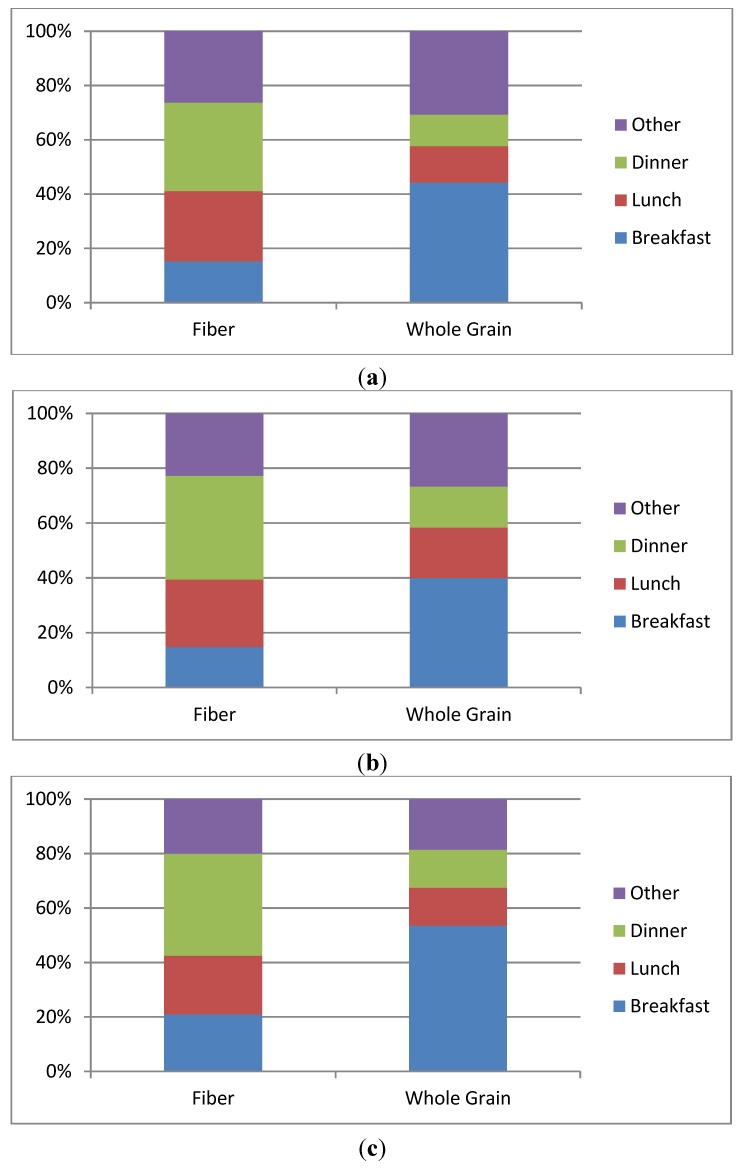
(**a**) Pattern of fiber and whole grain intakes by eating occasion for ages 4–18 years; (**b**) pattern of fiber and whole grain intakes by eating occasion for ages 19–50 years; (**c**) pattern of fiber and whole grain intakes by eating occasion for ages 51+ years.

Sample-weighted data were used in all statistical analyses, and all regression analyses were performed using SAS 9.2 with SUDAAN Release 11 (Research Triangle Institute, Research Triangle Park, NC, USA). Least square means (LSM) ± standard errors (SE) were calculated for fiber and WG intakes across the 2-year data cycles (Table 1 and Table 2). LSM ± SE for fiber and whole grain intakes by eating occasion was analyzed by age group for the combined 2001–2010 dataset (Figure 1). Table 3 and Table 4 include the LSM ± SE for fiber and WG intakes, respectively, by food group and the *p*-value for the 2001 through 2010 intake trend. A *p*-value of <0.05 was considered significant.

## 3. Results

Table 1 presents mean fiber intakes for children/adolescents and adults 19 to 50 y and 51 + y for each data cycle from 2001 through 2010 and for the 10-year period. The mean fiber intake for all children/adolescents for 2001–2010 was 13.2 (±0.1) g/day. The mean fiber intake for 2001–2010 for adults 19–50 y was 16.1 (±0.2) g/day and for adults 51+ was 16.1 (±0.2) g/day. There were significant increases in fiber intake (g/day) from 2001–2010 for children/adolescents 4 to 18 y and for adults 51+ y, but no change in fiber intake for adults 19–50 y from 2001–2010.

**Table 1:** Ten-year trends in fiber intakes by age.

Dietary Fiber (g/day)								
Age (y)	*n*	2001–2010	2001–2002	2003–2004	2005–2006	2007–2008	2009–2010	*p*-value for trend
4–18	14,973	13.2 ± 0.1	12.8 ± 0.2	13.2 ± 0.3	13.2 ± 0.3	13.0 ± 0.4	13.9 ±0.3	**0.016**
19–50	13,268	16.1 ± 0.2	16.2 ± 0.4	15.7 ± 0.4	15.7 ± 0.4	15.8 ± 0.5	17.0 ± 0.4	0.16
51+	11,541	16.1 ± 0.2	16.2 ± 0.5	15.2 ± 0.3	15.8 ± 0.3	16.1 ± 6	17.0 ± 0.2	**0.03**

Mean intakes of WG for all age groups for each data cycle from 2001 through 2010 and for the 10-year period are presented in Table 2. Mean WG intake for children/adolescents was 0.52 (±0.01) oz eq/day for 2001–2010. Mean WG intake for 2001–2010 for adults 19–50 y was 0.61 (±0.02) oz eq/day and for adults 51+ 0.86 (±0.02) oz eq/day. There were no significant changes in WG intake for any age group from 2001–2010.

**Table 2:** Ten-year trends in whole grain intakes by age.

Whole Grain (oz eq/day)								
Age (y)	*n*	2001–2010	2001–2002	2003–2004	2005–2006	2007–2008	2009–2010	*p*-value for trend
4–18	14,973	0.52 ± 0.01	0.56 ± 0.02	0.48 ± 0.03	0.48 ± 0.03	0.51 ± 0.02	0.55 ± 0.02	0.913
19–50	13,268	0.61 ± 0.02	0.62 ± 0.04	0.53 ± 0.03	0.62 ± 0.05	0.56 ± 0.04	0.72 ± 0.04	0.094
51+	11,541	0.86 ± 0.02	0.88 ± 0.05	0.77 ± 0.04	0.90 ± 0.05	0.84 ± 0.06	0.88 ± 0.04	0.587

The pattern of fiber and WG intakes by age and eating occasion is illustrated in Figure 1. For all age groups, fiber intake was highest at dinner. The contribution of dinner to total fiber intakes were 32% for children/adolescents 4–18 y, 38% for adults 19–50 y and 37% for adults 51+ y. Whole grain intake was highest at breakfast for all age groups. The contribution of breakfast to total WG intakes were 44% for children/adolescents, 39% for adults 19–50 y and 53% for adults 51+ y. Other eating occasions (snacks) make a significant contribution to intakes of both fiber and WG for all age groups. Snacks contributed ≥20% of total fiber intakes for the entire study population. The contribution of snacks to WG intakes were 31% for children/adolescents, 26% for adults 19–50 y and 19% for adults 51+ y.

Total dietary fiber intakes from various food groups and trends in intakes for 2001–2010 for the study population by age group are presented in Table 3. The main food groups contributing to total dietary fiber intake for children/adolescents were vegetables (16.6%), grain mixtures (16.3%), other foods (15.8%) and fruits (11.3%). The combined contribution of these food groups to total fiber intake for 2001–2010 was 60%. There were significant increases for 2001–2010 for fiber from quick breads, grain mixtures and fruits and a significant decrease in fiber from pastas among children/adolescents. For adults 19+ y, the main sources of total dietary fiber were vegetables (22.6%), other foods (14.3%), grain mixtures (12.0%) and fruits (11.1%). The combined contribution of these food groups to total fiber intake for 2001–2010 was 60%. There were significant increases for 2001–2010 for fiber from hot cereals/rice, RTEC, grain mixtures and fruits and a significant decrease in fiber from pastas for adults 19+. Ready-to-eat cereals contributed 6.3% of total dietary fiber for children/adolescents and 4.8% for adults 19+ y for 2001–2010.

**Table 3:** 10 year-trends in fiber food sources for children/adolescents (4–18 y) and adults (19+ y). RTE, ready-to-eat.

Food Category	2001–2010 Intake (g/d)	% Fiber Contribution	2001–2002 Intake (g/d)	2009–2010 Intake (g/d)	*p*-value for Trend
**Children/Adolescents *n* = 14,973**					
Yeast breads/rolls	1.2	10.5	1.2	1.4	0.88
Quick breads	0.4	2.7	0.3	0.4	**0.002**
Cakes/cookies/pies/pastries	0.6	5.2	0.6	0.6	0.40
Crackers and salty grain snacks	0.9	6.9	1.0	0.9	0.18
Pancakes/waffles/French toast/crepes	0.2	2.0	0.2	0.3	0.05
Pastas	0.08	0.6	0.2	0.08	**0.001**
Hot cereals/rice	0.2	1.4	0.2	0.2	0.88
RTE cereals	0.9	6.3	1.0	0.9	0.45
Grain mixtures/frozen plate meals/soups/meat substitutes	2.3	16.3	1.7	2.6	**<0.0001**
Dry beans/peas/legumes/nuts and seeds	0.8	4.4	0.3	0.3	0.22
Fruits	1.6	11.3	1.4	1.9	**<0.0001**
Vegetables	2.1	16.6	2.2	1.9	**0.02**
All other foods	1.9	15.8			
Total	13.2	100			
**Adults *n* = 24,809**					
Yeast breads/rolls	1.6	11.9	1.8	1.7	0.48
Quick breads	0.6	3.5	0.6	0.6	0.52
Cakes/cookies/pies, pastries	0.6	4.3	0.6	0.6	0.54
Crackers and salty grain snacks	0.7	4.6	0.8	0.7	0.18
Pancakes/waffles/French toast/crepes	0.1	0.9	0.1	0.1	0.44
Pastas	0.09	0.5	0.2	0.06	**0.009**
Hot cereals/rice	0.4	2.3	0.3	0.4	**0.01**
RTE cereals	0.9	4.8	1.0	1.1	**0.023**
Grain mixtures/frozen plate meals/soups/meat substitutes	2.0	12.0	1.5	2.0	**0.001**
Dry beans/peas/legumes/nuts and seeds	1.6	7.2	1.7	1.7	0.74
Fruits	2.0	11.1	1.9	2.4	**<0.0001**
Vegetables	3.4	22.6	3.7	3.4	0.16
All other foods	2.1	14.3			
Total	16.1	100			

Whole grain intakes from various food groups and trends in intakes for 2001–2010 for the study population are presented in Table 4. Major WG sources for children/adolescents included RTEC (31%), yeast breads/rolls (21%) and crackers and salty grain snacks (21%). The combined contribution of these food groups to WG intake was 73% for children/adolescents. The 10-year trends in intake were a significant increase in WG from yeast breads/rolls, quick breads, pancakes/waffles/French toast/crepes and grain mixtures. Significant decreases in WG from cakes/cookies/pies/pastries, RTEC and crackers and salty grain snacks were reported for 2001–2010 among children/adolescents. The main sources of WG for adults 19+ were yeast breads/rolls (27%), RTEC (23%) and pastas/cooked cereals/rice (21%), which comprised 71% of the total WG intake. There were significant increases in WG intakes from quick breads, pastas/cooked cereals/rice and grain mixtures for adults 19+ from 2001–2010. There was no change in WG intake from RTEC among adults for the 10-year period.

**Table 4:** 10-year trends in whole grain food sources for children/adolescents (4–18 y) and adults (19+ y).

Food Category	2001–2010 Intake (oz eq/d)	% WG contribution	2001–2002 Intake (oz eq/d)	2009–2010 Intake (oz eq/d)	*p*-value for Trend
**Children/Adolescents *n* = 14,973**					
Yeast breads/rolls	0.11	21	0.10	0.14	**0.01**
Quick breads	0.01	2	0	0.01	**0.02**
Cakes/cookies/pies, pastries	0.03	6	0.03	0.02	**0.004**
Crackers and salty grain snacks	0.11	21	0.11	0.11	0.45
Pancakes/waffles/French toast/crepes	0.03	6	0.02	0.04	**0.003**
Pastas/cooked cereals/rice	0.06	11	0.07	0.06	0.24
RTE cereals	0.16	31	0.22	0.14	**0.0003**
Grain mixtures/frozen plate meal/soups/meat substitutes	0.01	2	0	0.02	**0.003**
All other foods	0	0			
Total	0.52	100			
**Adults *n* = 24,809**					
Yeast breads/rolls	0.19	27	0.21	0.22	0.23
Quick breads	0.02	3	0.01	0.03	**0.01**
Cakes/cookies/pies/pastries	0.03	4	0.02	0.02	0.38
Crackers and salty grain snacks	0.12	17	0.14	0.12	0.28
Pancakes/waffles/French toast/crepes	0.02	3	0.02	0.02	0.56
Pastas/cooked cereals/rice	0.15	21	0.13	0.18	**0.003**
RTE cereals	0.16	23	0.18	0.17	0.4
Grain mixtures/frozen plate meals/soup/meat substitutes	0.01	1	0	0.01	**0.023**
All other foods	0.01	1			
Total	0.71	100			

## 4. Discussion

Mean intakes of fiber reported by NHANES 2001–2010 are well below (less than half) the current recommendations for all age groups. Our results are similar to reported fiber intakes in both the Institute of Medicine Dietary Reference Intakes Report [3] and the Dietary Guidelines for Americans, 2010 [1]. Mean fiber intakes reported in this study are close to values recently reported for U.S. adults by Grooms *et al.* [24] of 15.7–17.0 g/day. Reicks *et al.* [4] recently reported mean fiber intakes in the U.S. of 17.0 g/day for adults and 13.6 g/day for children/adolescents. The 10-year trends in mean fiber intake show an increase in fiber intake from 2001–2010 for children/adolescents age 4 to 18 y and adults aged 51+ y. There was no increase in fiber intake for adults aged 19 to 50 y. The absolute amount of mean fiber intake increases from 2001–2010 for children/adolescents was 1.1 g/day and for adults 51+ y 0.8 g/day. Although these increases are statistically significant, mean intakes remain below recommendations and illustrate a lack of change in fiber intake over time. A similar lack of change in fiber intake was reported for NHANES data from 1999–2008 among adults aged 18 and older [25].

Mean intakes of WG are also well below (less than one third) the recommendations for all age groups. The reported mean WG intakes in this study are similar to previous reports of 0.57 oz eq/day for children/adolescents [4], 0.63 oz eq/day for adults aged 19 to 50 y and 0.77 oz eq/day for adults aged 51+ y [10]. There were no changes in the 10-year trends in mean WG intake for any age group. In light of recent public health recommendations to increase WG intake [1,6] and increased availability of WG products in the market place [26], this lack of change is disappointing.

The present analysis reveals that the patterns of intake of dietary fiber and WG differ throughout the day. Dietary fiber intake is highest at dinner for all age groups (≥ one-third of total intake). Intake of WG is highest at breakfast, contributing 44% and 39% of total intake for children/adolescents and adults 19–50 y, respectively. For adults 51+ y, WG intake at breakfast was 53% of the total intake. These differences in pattern of intake for fiber and WG are likely due to differences in food sources consumed at breakfast and dinner. Snacks make a significant contribution to intakes of both fiber and WG (≥19% or more of total intake). Eating frequency, including snacks, has been reported to be positively related to fiber intake [27].

Food sources that were major contributors to fiber intake for children/adolescents were vegetables, grain mixtures, other foods and fruit, which contributed 60% of total fiber intake. There was an increase in fiber intake from quick breads, grain mixtures and fruit among children/adolescents from 2001–2010. Major fiber sources for adults aged 19+ y were vegetables, other foods, grain mixtures, yeast breads/rolls and fruit, which contributed 72% of the total intakes. There were increased intakes of fiber from hot cereals, rice, RTEC, grain mixtures and fruit among adults from 2001 to 2010. All age groups decreased intake of fiber from pastas from 2001 to 2010. Previous analyses of NHANES data reported similar top sources of dietary fiber. Keast *et al.* [14] and Reicks *et al.* [4] both reported that the top sources of fiber for children and adolescents were fruit, yeast bread/rolls and vegetables. Previous reports of top sources of fiber for adults were yeast bread/rolls and fruit [15].

Major sources of WG were somewhat different from fiber sources for all age groups. For children/adolescents, the top sources of WG were RTEC, yeast breads/rolls and crackers and salty snacks. For adults, major food group sources of WG were yeast breads/rolls, RTEC and pastas/cooked cereals/rice. Children/adolescents had an increase in WG intake from yeast breads/rolls, quick breads, pancakes/waffles/French toast/crepes and grain mixtures from 2001 to 2010. Intakes of WG from cakes/cookies/pie/pastries, RTEC and crackers and salty grain snacks decreased for children/adolescents from 2001 to 2010. The 10-year trend in WG intake for adults was increased intake from quick breads, pastas/cooked cereals/rice and grain mixtures. There was no change in WG intake from RTEC for adults from 2001–2010. Breads and cereals are the major food sources of WG in the diets of U.S. children/adolescents and adults [4,16]. This study reports an increase in WG intake from breads for the U.S. population from 2001–2010.

The present study reveals that dietary fiber and WG are consumed during different parts of the day and from different food groups. Fiber intake is highest at dinner, presumably when calorie intake is also highest. Vegetables and fruit are a major source of fiber for all age groups, but do not contribute to WG intake. Ready-to-eat cereal, commonly consumed at breakfast, was a major source of WG for all ages along with breads and grain mixtures. Intakes of WG from both breads and grain mixtures increased for all ages over the 10-year period. Ready-to-eat cereals in the marketplace differ in WG and fiber content, and there may be consumer confusion regarding the fiber and WG content of foods [4,28,29,30]. This may be a contributing factor to the low intakes of both dietary fiber and WG reported here and by others. Intakes of WG were highest at breakfast, when fiber intake was lower for all age groups. Consumer education to encourage the selection of RTEC with higher fiber and WG content is one step that could increase intakes for the U.S. population. Our data also reveal a significant contribution of snacks to both intakes of dietary fiber and WG. Increasing availability and selection of healthy snack products with higher contents of both fiber and WG would be another option to increase population intakes.

The limitations to this study include the cross-sectional design of NHANES, which does not allow causal inferences. Furthermore, a single 24-h dietary recall may not reflect the usual dietary pattern of participants and may under- or over-report food intake. However, NHANES is a large observational study of a nationally representative sample of the U.S. population that allows the assessment of numerous outcomes. Additional limitations include potentially underestimating fiber intake due to analytical methods that may not detect isolated/synthesized fibers, and current databases may not include WG products recently introduced into the marketplace and, thus, also underestimate WG intake.

## 5. Conclusions

In summary, total dietary fiber and WG consumption are well below current recommendations for all age groups. Ten-year trends in fiber intake increased for children/adolescents and adults 51+ y, but not in adults 19 to 50 y. There was no corresponding increase in WG intakes from 2001 to 2010. Dietary fiber and WG are consumed during different parts of the and from different food groups. Nutrition educators and policy recommendations should consider these differences in making dietary recommendations to consumers. Recommending cereals, breads and grain mixtures with higher contents of both dietary fiber and WG across all eating occasions, combined with consumer education, could increase intakes among the U.S. population.

## Abbreviations

oz eq: ounce equivalent
y: year
d: day
WG: whole grain
RTEC: ready-to-eat-cereals
